# Mitochondrial transplantation regulates antitumour activity, chemoresistance and mitochondrial dynamics in breast cancer

**DOI:** 10.1186/s13046-019-1028-z

**Published:** 2019-01-23

**Authors:** Jui-Chih Chang, Huei-Shin Chang, Yao-Chung Wu, Wen-Ling Cheng, Ta-Tsung Lin, Hui-Ju Chang, Shou-Jen Kuo, Shou-Tung Chen, Chin-San Liu

**Affiliations:** 1 Vascular and Genomic Center, Changhua Christian Hospital, Changhua, 50094 Taiwan; 2 Division of General Surgery, Department of Surgery, Changhua Christian Hospital, Changhua, 50094 Taiwan; 3 Department of Medicine, College of Medicine, China Medical University, Taichung, 40447 Taiwan; 4 Comprehensive Breast Cancer Center, Changhua Christian Hospital, Changhua, 50094 Taiwan; 5 Endoscopy & Oncoplastic Breast Surgery Center, Changhua Christian Hospital, Changhua, Taiwan; 6 Department of Neurology, Changhua Christian Hospital, Changhua, 50094 Taiwan; 7 Department of Chinese Medicine, China Medical University Hospital, Taichung, 40447 Taiwan; 8 School of Chinese Medicine, Graduate Institute of Chinese Medicine, Graduate Institute of Integrated Medicine, College of Chinese Medicine, Research Center for Chinese Medicine and Acupuncture, China Medical University, Taichung, 40447 Taiwan

**Keywords:** Mitochondrial transplantation, Pep-1, Apoptosis, Apoptosis-inducing factor, Oxidative stress, Chemoresistance, Mitochondrial dynamic, Metabolism

## Abstract

**Background:**

The transfer of whole mitochondria that occurs during cell contact has been found to support cancer progression. However, the regulatory role of mitochondria alone is difficult to elucidate due to the complex microenvironment. Currently, mitochondrial transplantation is an available approach for restoring mitochondrial function in mitochondrial diseases but remains unclear in breast cancer. Herein, effects of mitochondrial transplantation via different approaches in breast cancer were investigated.

**Methods:**

Whole mitochondria (approximately 10.5 μg/ml) were transported into MCF-7 breast cancer cells via passive uptake or Pep-1-mediated delivery. Fresh mitochondria isolated from homeoplasmic 143B osteosarcoma cybrids containing mitochondrial DNA (mtDNA) derived from health individuals (Mito) or mtDNA with the A8344G mutation (Mito^8344^) were conjugated with cell-penetrating peptide Pep-1 (P-Mito) or not conjugated prior to cell co-culture. Before isolation, mitochondria were stained with MitoTracker dye as the tracking label. After 3 days of treatment, cell viability, proliferation, oxidative stress, drug sensitivity to Doxorubicin/Paclitaxel and mitochondrial function were assessed.

**Results:**

Compared with P-Mito, a small portion of Mito adhered to the cell membrane, and this was accompanied by a slightly lower fluorescent signal by foreign mitochondria in MCF-7 cells. Both transplantations induced cell apoptosis by increasing the nuclear translocation of apoptosis-inducing factor; inhibited cell growth and decreased oxidative stress in MCF-7 cells; and increased the cellular susceptibility of both the MCF-7 and MDA-MB-231 cell lines to Doxorubicin and Paclitaxel. Mitochondrial transplantation also consistently decreased Drp-1, which resulted in an enhancement of the tubular mitochondrial network, but a distinct machinery through the increase of parkin and mitochondrial fusion proteins was observed in the Mito and P-Mito groups, respectively. Furthermore, although there were no differences in energy metabolism after transplantation of normal mitochondria, metabolism was switched to the energetic and glycolytic phenotypes when the mitochondria were replaced with dysfunctional mitochondria, namely, Mito^8344^ and P-Mito^8344^, due to dramatically induced glycolysis and reduced mitochondrial respiration, respectively. Consequently, transplant-induced growth inhibition was abolished, and cell growth in the Mito^8344^ group was even higher than that in the control group.

**Conclusion:**

This study reveals the antitumour potential of mitochondrial transplantation in breast cancer via distinct regulation of mitochondrial function.

**Electronic supplementary material:**

The online version of this article (10.1186/s13046-019-1028-z) contains supplementary material, which is available to authorized users.

## Background

Mitochondria are dynamic organelles whose dynamic properties not only govern energy generation but also control the gateway to cancer based on their comprehensive regulation of energy metabolism, calcium homeostasis, redox regulation and apoptosis. Mitochondrial dynamics are diverse in different cell types, and their function is determined by a delicate shift in the balance of morphological fusion and fission in cells to adapt to physiological stress. The role of mitochondrial dynamics in cancer development and progression is still complex and elusive because cancer cells are distinct from normal cells in terms of their ability to survive and proliferate despite their exposure to microenvironmental apoptotic stimuli, such as hypoxia, oxidative stress, nutrient deprivation and inflammation [1]. Recently, targeting mitochondrial dynamics has been considered a potential anticancer strategy. Inhibition of extensive mitochondrial fission has been demonstrated to suppress breast cancer metastasis [2], chemoresistance [1] and tumour stemness [3]. Moreover, mitochondrial dynamics have been shown to be involved in the mitigation of tumour cell escape from immune-mediated cellular destruction conducted by the tumour microenvironment [4]. Thus, the role of mitochondrial dynamics needs to be investigated during mitochondrial transfer (mitotransfer), which occurs frequently in cancer cell interactions with their surrounding environment [5].

Cancer cells are generally subjected to high levels of reactive oxygen species (ROS) compared to normal cells because ROS generation is augmented by mtDNA mutations and mitochondrial dysfunction [6]. In oestrogen-dependent breast cancer, ROS can induce cancer initiation and sustain cell cycle progression and death evasion through slow, sustained and moderate actions of ROS signalling that are induced by oestrogens and their various metabolites in females [7]. Elimination of the tumourigenic properties of ROS can be used for potential preventive and therapeutic approaches in human breast cancers and animal models of tumourigenesis in the early stage. Indeed, higher ROS production and decreased superoxide dismutases (SODs) and CAT activities have been observed in breast cancer patients, which supports the oxidative stress hypothesis in carcinogenesis [8]. SODs and catalase have been considered biomarkers for the early detection of breast cancer based on the detection of fat peroxidation in breast cancer [8]. Currently, antioxidants have been shown to promote mitotransfer in human mesenchymal stem cells [9], but this association in breast cancer remains unclear.

Mitotransfer between cells and in cell interactions has been widely studied recently in the regulation of cancer progression [10]. The horizontal transfer of whole mitochondria derived from mesenchymal stem cells and non-malignant cells in a co-culture system restores tumourigenic potential in mitochondrial DNA (mtDNA)-deficient skin cancer cells [11] and increases the invasiveness of bladder cancer cells [12], respectively. Intriguingly, the opposite result is observed in breast cancer cells received an artificial transfer of non-malignant mitochondria isolated from epithelial cells [13]. It means that the regulatory role of cancer mitochondria is tricky in cancer cells, especially in occurrence of cellular mitotransfer initiated by the synergistic or paracrine regulation from the peripheral environments [14]. Previously, we showed the feasibility of mitochondrial transplantation using a Pep-1-mediated delivery system to rescue mitochondrial function in a mitochondria-dependent manner and to decrease oxidative stress in mitochondrial diseases, including myoclonic epilepsy with ragged-red fibres (MERRF) [15–17]; mitochondrial encephalomyopathy, lactic acidosis, and stroke-like episodes (MELAS) [18]; and Parkinson’s disease [19]. In contrast to the passive endocytosis of foreign mitochondria [20], Pep-1-mediated mitochondrial delivery (PMD) facilitates the uptake of mitochondria by initiating mitochondrial crossing of the cell membrane and promotes mitochondrial fusion with a consequent decrease in mitochondrial fission in diseased cells [15, 18]; however, the utility of PMD in the treatment of breast cancer, a mitochondrial disorder, remains unknown [21].

The present study offers another approach for investigating the mitochondria-targeted treatment of breast cancer via mitochondrial transplantation. Compared to a previous pilot study [13], this study further compares the different routes of mitochondrial transplantation via passive uptake and Pep-1-mediated delivery in terms of their impacts on oxidative stress, chemoresistance, mitochondrial dynamics and energy metabolism in breast cancer cell lines, and these effects have not been clarified to date. Moreover, by replacing normal mitochondria with dysfunctional mitochondria isolated from MERRF cybrid cells, we further examine mitochondria-dependent regulation in treated breast cells.

## Methods

### Cell culture

The human breast adenocarcinoma cell lines MCF-7 and MDA-MB-231 were purchased from BCRC (Bioresource Collection and Research Center) (Food Industry Research and Development Institute, Taiwan). MCF-7 cells were cultured in minimum essential medium (MEM) (Gibco/BRL, Life Technologies, Grand Island, NY, USA) with 2 mM L-glutamine and Earle’s BSS adjusted to contain 1.5 g/L sodium bicarbonate, 0.1 mM non-essential amino acids and 1 mM sodium pyruvate and supplemented with 0.01 mg/ml bovine insulin and 10% foetal bovine serum (FBS, Gibco/BRL). MCF-7 cells were incubated at 37 °C with 5% CO_2_. MDA-MB-231 cells were cultured in Leibovitz’s L-15 medium (Gibco/BRL) supplemented with 10% FBS, 1% penicillin-streptomycin (100 U/L penicillin G sodium and 100 mg/L streptomycin sulfate) (Gibco/BRL) and incubated at 37 °C without CO_2_. Mitochondrial donor cells from the homeoplasmic 143B osteosarcoma cybrid cell line that carried wild-type mitochondria derived from healthy individuals (C2) or mitochondria with mtDNA containing the A8344G mutation A > G derived from MERRF patients (B2) were gifts from Prof. Y.H. Wei [22]. Homeoplasmic 143B osteosarcoma cybrid cells were cultured in high-glucose DMEM (Gibco/BRL) supplemented with 10% FBS, 1% penicillin-streptomycin, 1 mM sodium pyruvate and 50 mg/ml uridine (Sigma-Aldrich, St. Louis, MO, USA) and incubated at 37 °C with 5% CO_2_. Cells were detached from tissue culture flasks by digestion with 0.25% trypsin and 0.02% EDTA (Gibco/BRL).

### Mitochondrial labelling

To track internalized mitochondria in treated cells, mitochondria were genetically encoded with green fluorescent protein (GFP) prior to isolation from the donor cells described in [17]. For transient transfection, mitochondrial donor cells were transfected using a PureFection transfection reagent (System Biosciences, Mountain View, CA, USA) with pAC-GFP-mito plasmid DNA (Clontech, Palo Alto, CA, USA) at a reagent to DNA ratio of 1.5:1 ratio per the manufacturer’s instructions. After 36 h, the transfected cells were placed in normal medium for 4 h and treated with G418 (0.6 mg/ml; Sigma-Aldrich) for clonal selection.

### Mitochondrial isolation and delivery

The process of mitochondrial isolation and Pep-1-mediated mitochondrial delivery have been described in our previous studies as well as viability check of isolated mitochondria [16–18]. Briefly, the donor cells were harvested and then washed once in PBS. A total of 2 × 10^7^ cells were pelleted for mitochondrial isolation according to the manufacturer’s protocol included in the mitochondria isolation kit for mammalian cells (Thermo Fisher Scientific, Carlsbad, CA, USA). Cells were homogenized using a glass Dounce tissue grinder in isolation reagent supplemented with proteinase inhibitor (EMD Millipore, Billerica, MA, USA) on ice. After centrifugation, the supernatant was discarded, and the concentration of freshly isolated mitochondria was quantified by assessing total protein content using a standard curve of bovine serum albumin with the bicinchoninic acid (BCA) kit assay (Pierce, Rockford, IL, USA) [23]. An average of 2 × 10^7^ donor cells can isolate about 780 ± 89.7 μg mitochondria in total. Isolated mitochondria (approximately 105 μg, concentration of 0.26 μg/μL) were conjugated with Pep-1 peptide (0.06 mg, concentration of 0.05 mM, Anaspec, San Jose, CA, USA) (P-Mito) or not conjugated (Mito). To form the P-Mito complex, mitochondria were conjugated by gentle mixing and allowed to stand at room temperature for 10 min. Mitochondria were then delivered into indicated breast cancer cell lines that were seeded at 5 × 10^5^ cells in 10-cm dishes with the regular medium, and mixed by gentle pipetting without turbulence. After treatment with Mito or P-Mito for 48 h, the recipient cells were washed twice with PBS and cultured with regular medium. All experiments except the related evaluation of mitochondrial internalization were executed after 3 days of recovery.

### Detection of mitochondrial internalization and apoptosis

After the delivery of GFP-tagged mitochondria (Mito^GFP^) with or without Pep-1 modification was complete, the internalization of foreign mitochondria was assessed immediately by flow cytometry (Beckman Coulter FC500 Cytometer, Canton, MA, USA). At same time, three-dimensional scanning of the z-axis using a laser scanning confocal microscope (FLUOVIEW FV1200 Biological Confocal Laser Scanning Microscope, Olympus, Tokyo, Japan) was utilized to analyse the distributions and interactions of the foreign mitochondria in the host cells. To distinguish the innate mitochondria of the host cells, these mitochondria were labelled first with mitochondria-specific red dye (100 nM MitoTracker Red CMXRos, Life technologies) for 20 min in a CO_2_ incubator before the treatments. The estimation of mitochondrial internalization (Mito^GFP^) was repeated three times and interaction of foreign and innate mitochondria was monitored for each of the five field regions. Furthermore, the time-dependent effect in the rate of apoptosis was recorded over a period of 24 h at 10-min intervals using a laser scanning confocal microscope to measure apoptotic cells that were stained with propidium iodide (PI) (final concentration, 2.5 μg/ml; Life Technologies, Thermo Fisher Scientific, Waltham, MA, USA). Combinatorial use of bright field cell imaging was carried out to identify and monitor the subpopulations of PI-positive apoptotic cells in the total or GFP-positive cell population. The data calculation was obtained by counting percentage of fluorescence-positive cells in three unintentionally chosen fields of vision from three independent experiments.

### Cell proliferation

Cell proliferation was measured using the cell proliferation reagent WST-1 (Roche Applied Sciences, Mannheim, Germany). A total of 7.5 μl of WST-1 (5%) was added to 150 μl of fresh medium per well in 24-well plates and incubated for 1 h at 37 °C. The plate was shaken thoroughly for 1 min, and the supernatants were collected by centrifugation to measure the optical density (OD) at 450 nm with a plate reader (CLARIOstar microplate reader, BMG LABTECH, GmbH, Offenburg, Germany).

### Reactive oxygen species (ROS) production

ROS production was analysed comprehensively by individually measuring total ROS using the 2,7-dichlorofluorescein diacetate (DCFH-DA, Life Technologies) probe, mitochondrial superoxide production using the MitoSox Red (Life Technologies) probe and superoxide using the dihydroethidium probe (DHE, Life Technologies). DCFH-DA working solution was added directly to the medium to reach 10 μM and then incubated at 37 °C for 15 min in the dark; 50 nM MitoSox Red and 20 μM DHE were used to individually stain cells for 30 min at 37 °C in the dark. All stained cells were washed twice with PBS, resuspended in PBS and kept on ice for immediate detection by flow cytometry (FC500, Beckman Coulter) at the optimal excitation/emission wavelengths (DCFH-DA at 485/535 nm; MitoSox Red at 510/580 nm; DHE at 480/576 nm). Data were consistently analysed using FlowJo software (Ashland, OR, USA).

### Drug sensitivity of doxorubicin and paclitaxel

Cells were seeded 1 × 10^4^ cells/well into 96-well culture plates with culture medium (200 μl/well) containing indicated concentrations of Doxorubicin (range, 0.8 μM to 1.4 μM) or Paclitaxel (range, 5 μM to 30 μM). After 72 h of incubation, cell viability was measured by WST-1 assay as described below. The lethal concentration 50 (LC50) value, the drug concentration at which 50% of the cells were killed by the drug, was used as a measure of sensitivity.

### Analysis of mitochondrial morphology

Cells were seeded to a chamber slide after 3 days of treatment (μ-Slide 8 well, Ibidi), stained with a mitochondrial potential-independent dye, MitoTracker Deep Red (25 nM, Life Technologies), and incubated at 37 °C for 30 min. After the excess dye was removed, cells were washed with PBS, fixed with 4% paraformaldehyde (Sigma-Aldrich) and imaged using an Olympus FluoView FV 1200 Confocal Microscope. Subtyping of mitochondrial morphology was quantified using an automatic classification system according to Peng et al. [24]. After semi-automatic segmentation of cell micrographs, mitochondria were classified into six distinct subtypes (small globe, swollen globe, straight tubule, twisting tubule, branch tubule and loop) using automatic classification software. The proportion of elongated mitochondria was calculated by summing the branch tubule and loop mitochondrial populations. The proportion of tubular mitochondria was calculated by summing the straight tubule and twisting tubule mitochondrial populations. The proportion of fragmented tubular mitochondria was calculated by summing the small globe and swollen globe mitochondrial populations. Approximately 150–250 mitochondria from approximately 3–5 cells in each image from three independent areas per group were counted.

### Copy numbers of mitochondrial DNA

Fifty nanograms of total DNA was used to amplify the nicotinamide adenine dinucleotide dehydrogenase subunit 1 (ND1) gene (mtDNA-encoded) and the β-actin gene (nuclear DNA-encoded, used as an internal control) with specific primer pairs by quantitative PCR with LightCycler® Systems (Roche Applied Sciences) and FastStart DNA Master SYBR Green I kit (Roche Applied Sciences). The relative mtDNA copy number was determined by normalizing the intersecting points on the quantitative PCR curves between the ND1 and β-actin genes using RelQuant software (Roche Applied Science).

### Mitochondrial mass

To analyse the mitochondrial mass, 0.1 μM 10-N-nonyl acridine orange (NAO) (Sigma-Aldrich) was used, and incubations were carried out for 30 min in regular medium. After washes in PBS, fluorescence was analysed by flow cytometry at an excitation wavelength of 485 nm and emission wavelength of 520 nm. Autofluorescence of the control cells was examined to confirm the staining level at the same time, and the value of the fluorescence intensity was expressed relative to that of the control cells.

### Western blot analysis

Whole cell lysates were prepared using RIPA lysis buffer (1% deoxycholic acid, 1% Triton X-100, 0.1% SDS, 250 mM NaCl and 50 mM Tris–HCl, pH 7.5) with sonication. Cytosolic and nuclear fractions were extracted with a Nuclear/Cytosolic Fractionation kit (BioVision, Mountain View, CA, USA). Whole cell lysates or nuclear/cytosolic fractionations were clarified by centrifugation, and the protein concentrations were determined with the Pierce BCA Protein Assay kit (Thermo Scientific). The samples were boiled in loading buffer, and approximately 30 μg of protein was subjected to electrophoresis on 10% or 12% Mini-PROTEAN TGX Stain-Free Gels (Bio-Rad, Hercules, CA, USA). After electrophoresis, proteins were transferred to polyvinylidene difluoride (PVDF) membranes using the Trans-Blot Turbo RTA Mini PVDF transfer kit (Bio-Rad) and a Trans-Blot Turbo Blotting system (Bio-Rad) according to the manufacturer’s instructions. Membranes were then blocked in BlockPRO™ Blocking Buffer (Visual Protein Biotechnology, Taipei, Taiwan) for 1 h at room temperature and probed with monoclonal antibodies at their indicated dilutions for cell death-related proteins including cytochrome c (Cyto c, Cell Signaling Technology, Danvers, MA, USA), cleaved caspase-9 (Cell Signaling Technology), Phospho-p53 (Ser15, p-p53, Cell Signaling Technology) and apoptosis-inducing factor (AIF) (Abcam, Cambridge, MA, USA) and for mitochondrial dynamic proteins including optic atrophy 1 (OPA1, Novus Biologicals, Littleton, CO, USA), Mitofusin 2 (MFN2, Sigma-Aldrich), dynamin-related protein 1 (Drp-1, Novus Biologicals), PTEN-induced putative kinase 1 (PINK-1, Abcam) and Parkin (Abcam). Membranes were probed with HRP-conjugated secondary antibodies, and bound antibodies were visualized and quantified using the Chemiluminescence Western HRP Substrate (EMD Millipore, Billerica, MA, USA) and the FUSION SL image acquisition system (Viber Lourmat, Marne-la-Vallee, France).

### Seahorse XF24 extracellular flux analyzer

Cells were seeded in a XF24 microplate (1 × 10^4^/per well) and cultured with normal medium for 16–18 h before mitochondrial function assays, according to the manufacturer’s recommended protocol provided with the Seahorse XF24 Extracellular Flux Analyzer (Seahorse Bioscience, North Billerica, MA, USA). Cell media were replaced with conditioned medium (high-glucose DMEM without FBS or sodium bicarbonate) and incubated at 37 °C without CO_2_ for 1 h before completion of the probe cartridge calibration. The basal oxygen consumption rate (OCAR) and extracellular acidification rate (ECAR) were measured with the Seahorse XF24 Flux Analyzer. Measurements were performed after injections of three compounds that affect bioenergetics: 1 μM Oligomycin (Sigma-Aldrich), 0.3 μM carbonyl cyanide 4-(trifluoromethoxy)phenylhydrazone (FCCP, Sigma-Aldrich) and 1 μM Rotenone (Sigma-Aldrich). Upon completion of the Seahorse XF24 Flux analysis, cells were lysed to calculate protein concentrations with a BCA assay (Thermo Scientific). The result was normalized to the protein OD value of the corresponding well.

### Statistical analysis

GraphPad Prism or Microsoft Excel 2010 were used to generate all graphs and perform all statistical analyses. A minimum of three independent replications of each experiment were performed. Student’s t-test was employed to test statistical significance, and *p* values less than 0.05 were judged to indicate statistical significance.

## Results

### Mitochondrial transplantations via passive uptake and Pep-1-mediated delivery

**Fig. 1:**
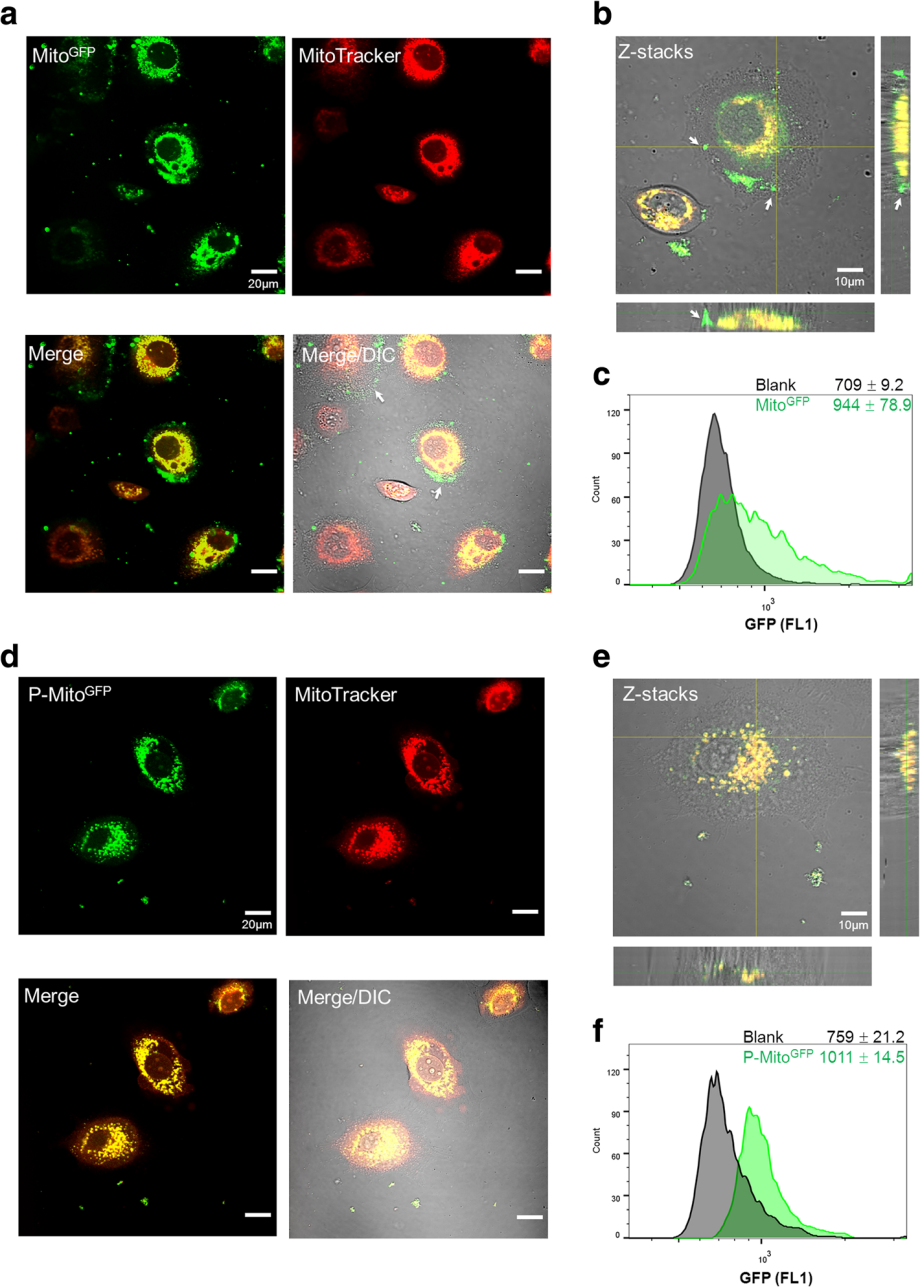
Expression of foreign mitochondria tagged with green fluorescent protein (Mito^GFP^) in MCF-7 human breast cancer cells pre-stained with MitoTracker Red. Internalization of Mito^GFP^ (**a-c**) or Pep-1-labelled Mito^GFP^ (P-Mito^GFP^) (**d-f**) was observed by confocal microscopy with different colour labels combined with the differential interference contrast (DIC)/bright field channel after 2-day treatments. The colocalization of foreign (green) and innate mitochondria (red) is shown in merged images (**a, d**) and Z-stacks (**b, e**), respectively. The white arrows indicate adhesion of Mito^8344^ to the outer cell membrane and entry failure (**a, b**). The quantification of mitochondrial internalization was performed by flow cytometry and is represented as the median fluorescence intensity of GFP with the standard deviation (**c, f**). Blank indicates the cell background of each group before treatment

After a 48-h co-culture of GFP-labelled mitochondria (Mito^GFP^, Fig. 1a-c) or Pep-1-modified Mito^GFP^ (P-Mito^GFP^, Fig. 1d-f) with MCF-7 breast cancer cells whose mitochondria were pre-stained with MitoTracker Red, the foreign mitochondria (green) were clearly internalized in both treatment groups and translocated into the host-cell mitochondria (red), as indicated by the yellow signals shown in Fig. 1a and d. Moreover, the combination of one transmitted light contrast technique (DIC) with fluorescence and z-axis scanning confocal microscopy confirmed the colocalization of foreign and innate mitochondria in the cells (Fig. 1b and e) and further revealed that a portion of Mito^GFP^ preferentially remained at the cell membrane (indicated by white arrows, Fig. 1a and b), in contrast to P-Mito^GFP^ (Fig. 1d and e). The labelling efficiency of P-Mito^GFP^ (fluorescence intensity relative to blank, Fig. 1f) was slightly higher than that of Mito^GFP^ (Fig. 1c), as detected by flow cytometry.

### Mitochondrial transplantation initiates AIF-mediated apoptosis and suppresses cancer cell growth

**Fig. 2:**
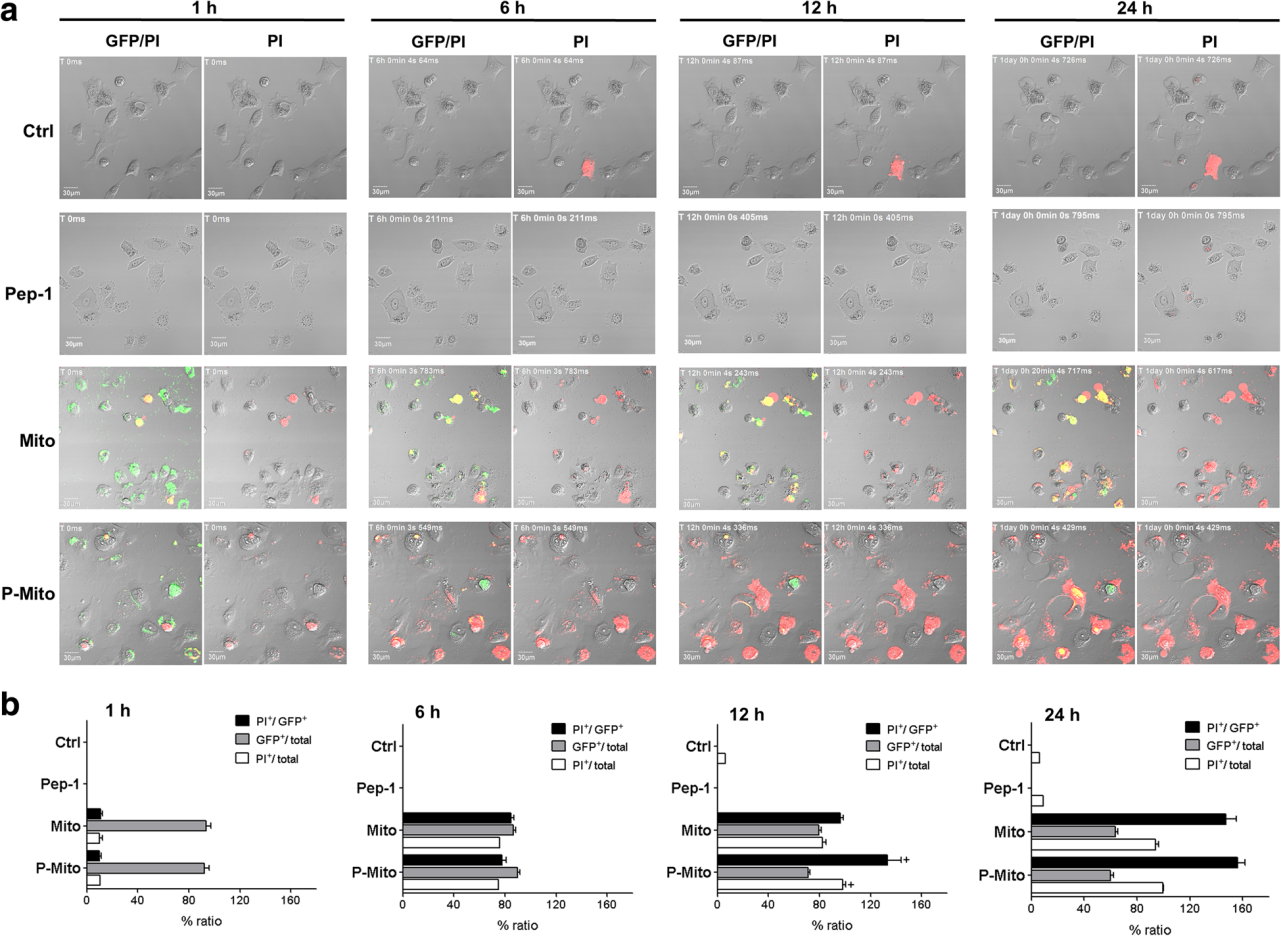
Occurrence tracking of apoptosis in MCF-7 cells during the internalization of foreign mitochondria. Continuous tracking of apoptosis using propidium iodide (PI)-incorporating medium in cells with internalized mitochondria (Mito^GFP^ or P-Mito^GFP^) over time was executed with 12-h video recordings from the same region (**a**). The occurrence and quantification of apoptosis normalized to the total or GFP-positive cell population, as well as GFP expression normalized to the total cell population, over time is shown at different time points, namely, 1, 6, 12 and 24 h (**b**). + *p* < 0.05, the differences between the Mito^GFP^ and P-Mito^GFP^ groups

Real-time tracking of apoptotic potency during the internalization process of Mito^GFP^ or P-Mito^GFP^ was executed by simultaneous co-staining with PI, a cell impermeable nuclear dye (Fig. 2). Approximately 80% of cells had a GFP-positive signal (green) (GFP^+^/total cell population) derived from Mito^GFP^ or P-Mito^GFP^ at the beginning of the 1–6 h treatment (Fig. 2b), and then, GFP fluorescence decayed with time (Fig. 2a). Apparent apoptosis of MCF-7 cells (red) was observed in cells that had internalized Mito^GFP^ or P-Mito^GFP^ after 6 h of treatment (PI^+^/GFP^+^ population, 85 ± 2.3% and 79 ± 3.5%) and there was no difference in the apoptotic incidence with respect to the total cells (PI^+^/total population) (Fig. 2b). After 12 h of treatment, the apoptotic cell populations (PI^+^/total population) in P-Mito group (94 ± 3.1%) was significantly higher than Mito group (82.3 ± 4.2%) and both of them were all over 90% after 24 h of treatment (Fig. 2b). It meant that the P-Mito induction of apoptotic potency was more potent than Mito.

**Fig. 3:**
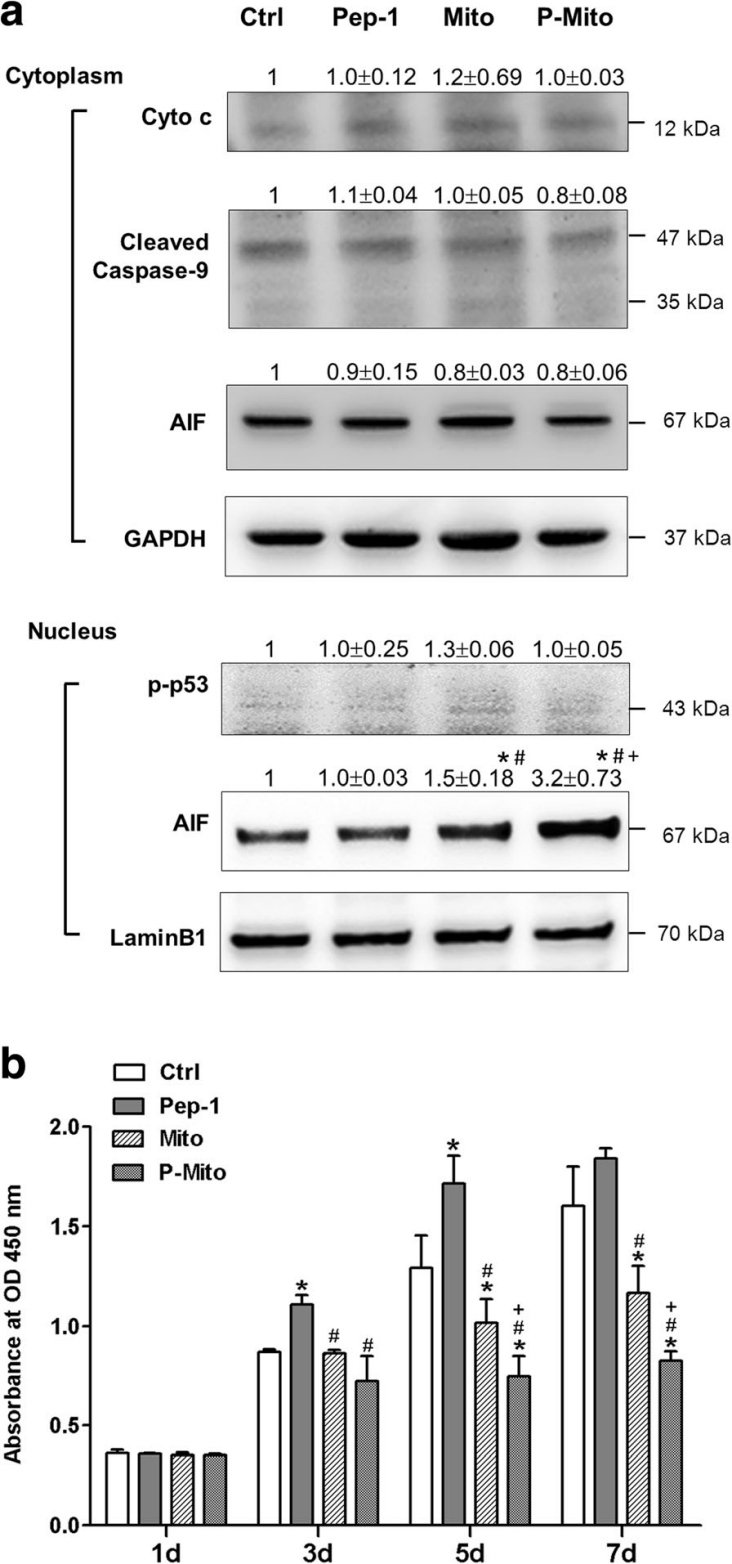
Expression of apoptosis-related proteins and cell viability in treated MCF-7 cells. Cytochrome c (Cyto c) and apoptosis-induced factor (AIF) proteins that were released from mitochondria and cleaved caspase-9 protein in the cytosol were analysed after 3-day treatments, as was the nuclear translocation of phosphorylated p53 (p-p53) and AIF (**a**). Simultaneously, cell viability was evaluated by WST-1 proliferation assay on days (d) 1, 3, 5 and 7 (**b**). * p < 0.05, difference relative to the control (Ctrl) group. # p < 0.05, difference relative to the Pep-1 group. + p < 0.05, difference between Mito and P-Mito groups

Mitochondrial transplantation of Mito and P-Mito significantly induced the nuclear translocation of AIF in MCF-7 cells after 3 days of treatment; this was especially notable for P-Mito, which was associated with a higher induction of nuclear translocation. There were no differences in the release of the other pro-apoptotic proteins including cytochrome c (Cyto c), cleaved caspase-9 and AIF, from the mitochondria to the cytosol or in the phosphorylation of p53 Serine 15 (p-p53) in the nucleus (Fig. 3a). Similar observations were made in MCF-7 cells after 3 days of treatment. Suppression of cancer cell growth with P-Mito treatment was more significant than that with Mito treatment. In contrast, treatment with Pep-1 alone did not affect cell apoptosis (Figs. 2 and 3a) but significantly promoted cell proliferation (Fig. 3b).

### Mitochondrial transplantation decreases oxidative stress and chemoresistance

**Fig. 4:**
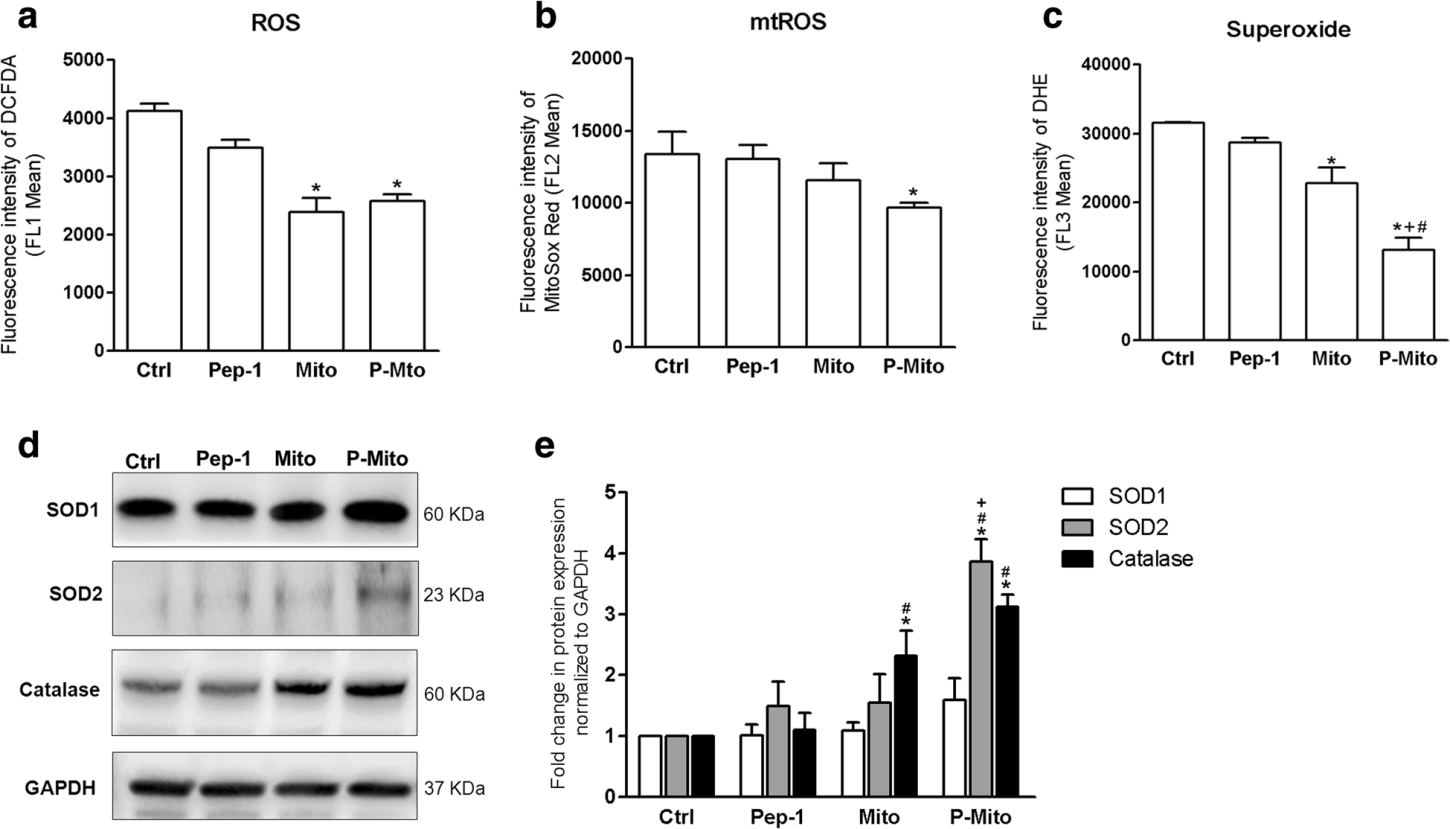
The occurrence of oxidative stress in treated MCF7 cells. The comprehensive analysis of reactive oxygen species (ROS) generation included total ROS (**a**), mitochondrial ROS (mtROS) (**b**) superoxide (**c**) and antioxidative enzymes, namely, superoxide dismutases (SODs) (copper/zinc superoxide dismutase SOD1 and manganese superoxide SOD2) and catalase (**d, e**) after 3 days of treatment. * *p* < 0.05, difference relative to the control (Ctrl) group. # *p* < 0.05, difference relative to the Pep-1 group. + *p* < 0.05, difference between the Mito and P-Mito groups

In both the Mito and P-Mito groups, after 3 days of treatment, there were significant decreases in ROS production in terms of total ROS (Fig. 4a) and superoxide (Fig. 4b), as detected by flow cytometric analysis of 2′,7′–dichlorofluorescin diacetate (DCFDA) and dihydroethidium (DHE) staining, respectively. The obvious reduction in mitochondrial ROS (mtROS) detected by MitoSox Red staining was only observed in the P-Mito group relative to the control group (Fig. 4c). The antioxidant enzyme catalase was dramatically and consistently increased in both the Mito and P-Mito groups, in contrast to the mitochondria-localized manganese superoxide dismutase (Mn-SOD, SOD2), which only showed a significant increase in the P-Mito group (Fig. 4c and d). Pep-1 affected neither ROS generation nor antioxidant enzymes (Fig. 4).

**Fig. 5:**
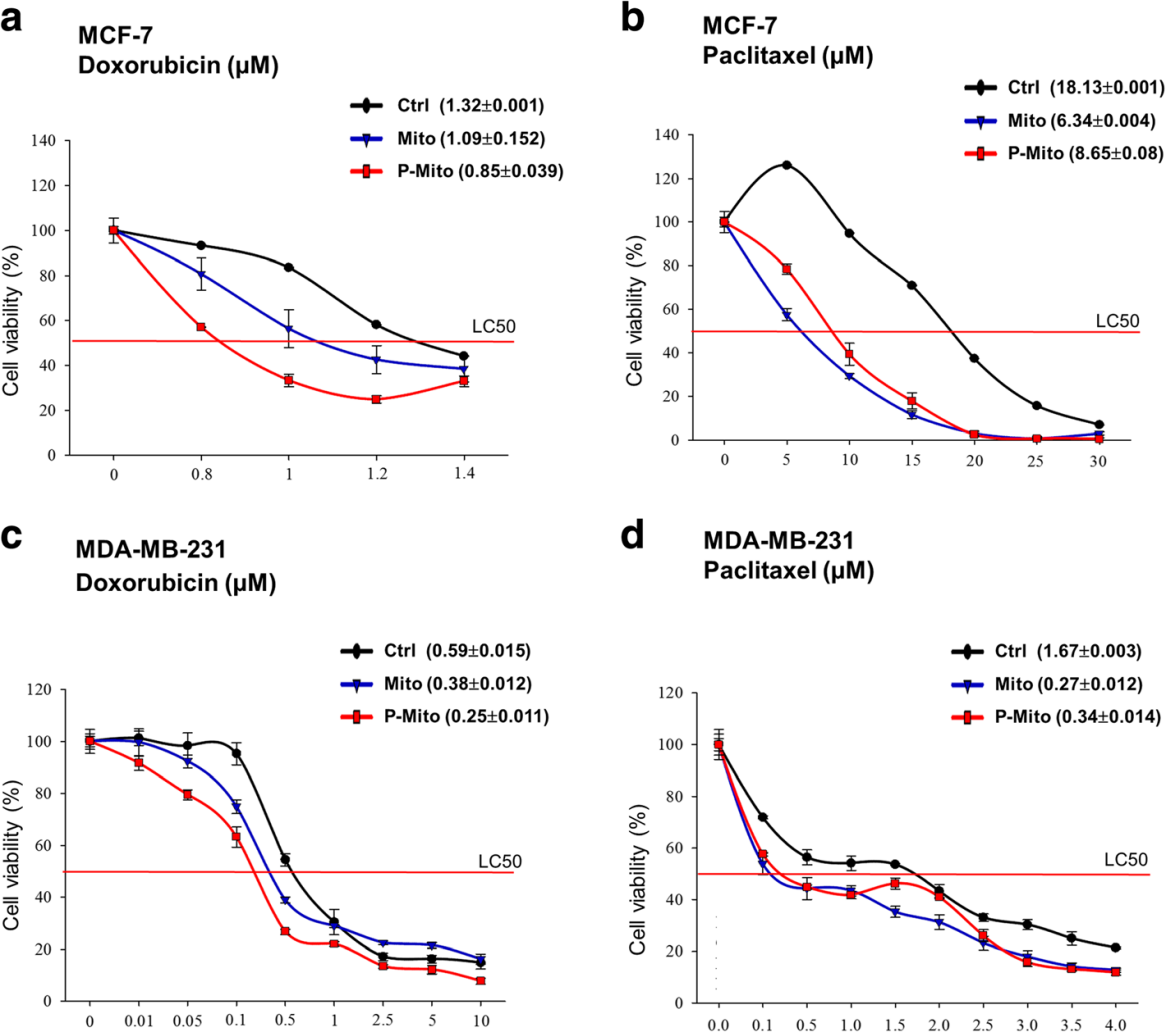
Changes in drug resistance via mitochondrial transplantation. The cellular viability assay was used to monitor the drug toxicity of Doxorubicin and Paclitaxel in both MCF7 (**a, b**) and MDAMB231 cells (**c, d**) after 3 days of treatment with Mito or P-Mito. Drug toxicity is expressed as the treatment concentration (μM) that is lethal to 50% of the cells (LC50) (Mean ± SD)

Comparisons of chemoresistance as defined with the lethal concentration (μM) 50 (LC50), value to two anticancer drugs (Doxorubicin and Paclitaxel) in two types of breast cancer cell lines (MCF7 and MDA-MB-231 cells) were performed under the Mito and P-Mito treatments (Fig. 5). The Mito and P-Mito treatments dramatically and effectively decreased resistance to the two drugs in both MCF-7 (Fig. 5a and b) and MDA-MB-231 cells (Fig. 5c and d) after 3 days of treatment. Moreover, the regulation of chemoresistance via mitochondrial transplantation was dependent on the different types of drugs. The increases in the sensitivity of both cell lines to Doxorubicin were higher for the P-Mito treatment than for the Mito treatment (LC50 of MCF-7 from 1.32 μM to 0.85 μM and 1.09 μM with P-Mito and Mito, respectively, Fig. 5a; LC50 of MDA-MB-231 from 0.59 μM to 0.25 μM and 0.38 μM for P-Mito and Mito, respectively, Fig. 5c); however, the increases in the sensitivity of both cell lines to Paclitaxel were comparable between the Mito and P-Mito treatments (LC50 of MCF-7 from 18.13 μM to 6.34 μM and 8.65 μM for Mito and P-Mito, Fig. 5b; LC50 of MDA-MB-231 from 1.67 μM to 0.27 μM and 0.34 μM for Mito and P-Mito, respectively, Fig. 5d).

### Mitochondrial transplantation increased parkin protein and decreased mitochondrial fragmentation as a consequence of mitochondrial fusion

**Fig. 6:**
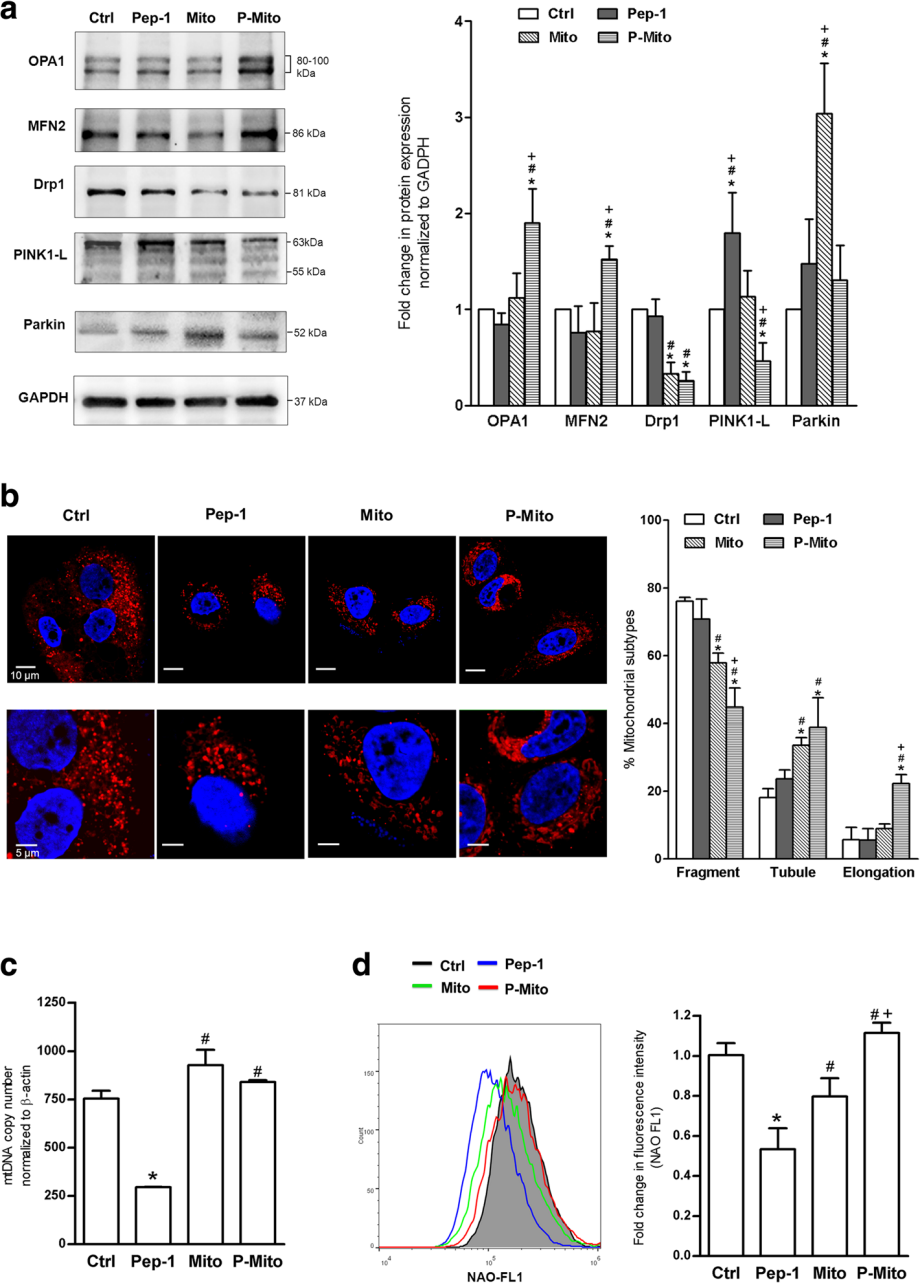
Expression of proteins involved in mitochondrial dynamics and biogenesis in treated MCF7 cells. After 3-day treatments, the levels of the mitochondrial dynamic-related proteins optic atrophy-1 (OPA1), Mitofusin 2 (MFN2), and dynamin-related protein 1 (Drp-1), as well as mitophagy-related proteins, namely, full-length PTEN-induced putative kinase 1 (PINK-1-L) and Parkin, were analysed and quantified (**a**). Mitochondrial morphology in terms of MitoTracker Red staining (**b**) was quantified with an automatic classification system to classify morphology into three distinct morphological subtypes, namely, fragmented, tubular and elongated (b, right panel). Mitochondrial biogenesis was evaluated by analysing the copy number of mitochondrial DNA (mtDNA) relative to that of the β-actin gene (**c**), and mitochondrial mass was measured with 10-N-nonyl acridine orange (NAO) staining and flow cytometry (**d**). * *p* < 0.05, difference relative to the control (Ctrl) group. # *p* < 0.05, difference relative to the Pep-1 group. + *p* < 0.05, difference between the Mito and P-Mito groups

While the Mito treatment significantly increased the parkin protein level relative to the control, P-Mito treatment enhanced the mitochondrial fusion protein OPA1 and MFN2 levels and decreased the full-length PINK1 (PINK1-L) protein level after 3 days of treatment in MCF-7 cells (Fig. 6a). Consistently, a significant decrease in mitochondrial fission protein Drp1 was observed with both treatments relative to the control (Fig. 6a). Pep-1 treatment dramatically increased PINK1-L protein compared with the other treatments (Fig. 6a). In the examination of mitochondrial morphology via staining with MitoTracker Red, extensive fragmentation of mitochondria was found in the control group compared to the Mito and P-Mito groups, with tubular networks and branch-like structures of mitochondrial morphology, respectively (Fig. 6b). Using an automatic classification software to classify the mitochondrial morphology of treated cells into three morphological subtypes, namely, fragmented, tubular and elongated, in the total population, we observed significant enhancement of the tubular subtype with a concomitant reduction in the fragmented subtype the in Mito and P-Mito groups (Fig. 6b, right panel). Moreover, the P-Mito group had more significant increases in mitochondrial elongation (Fig. 6b) and the mitochondrial amount than the Mito group (Fig. 6e) following an apparent inhibition of the fragmented subtype (Fig. 6b). This result, combined with the results of the western blot analysis (Fig. 6a), reflected the phenomenon of mitochondrial hyperfusion via enhancement of the elongated and branched morphology in the P-Mito group, in contrast to the intrinsic balance of mitochondrial fusion and fission in the Mito group. On the other hand, there was no significant difference in mitochondrial biogenesis revealed by the mtDNA copy number (Fig. 6c) or mitochondrial mass (Fig. 6d) in the Mito and P-Mito groups compared with the control group. Pep-1 treatment did not affect mitochondrial morphology (Fig. 6b) but dramatically inhibited mitochondrial biogenesis (Fig. 6c and d).

### Replacement of normal mitochondrial transplantation with dysfunctional mitochondria abolishes the original inhibition of cancer cell growth via distinct metabolic reprogramming

**Fig. 7:**
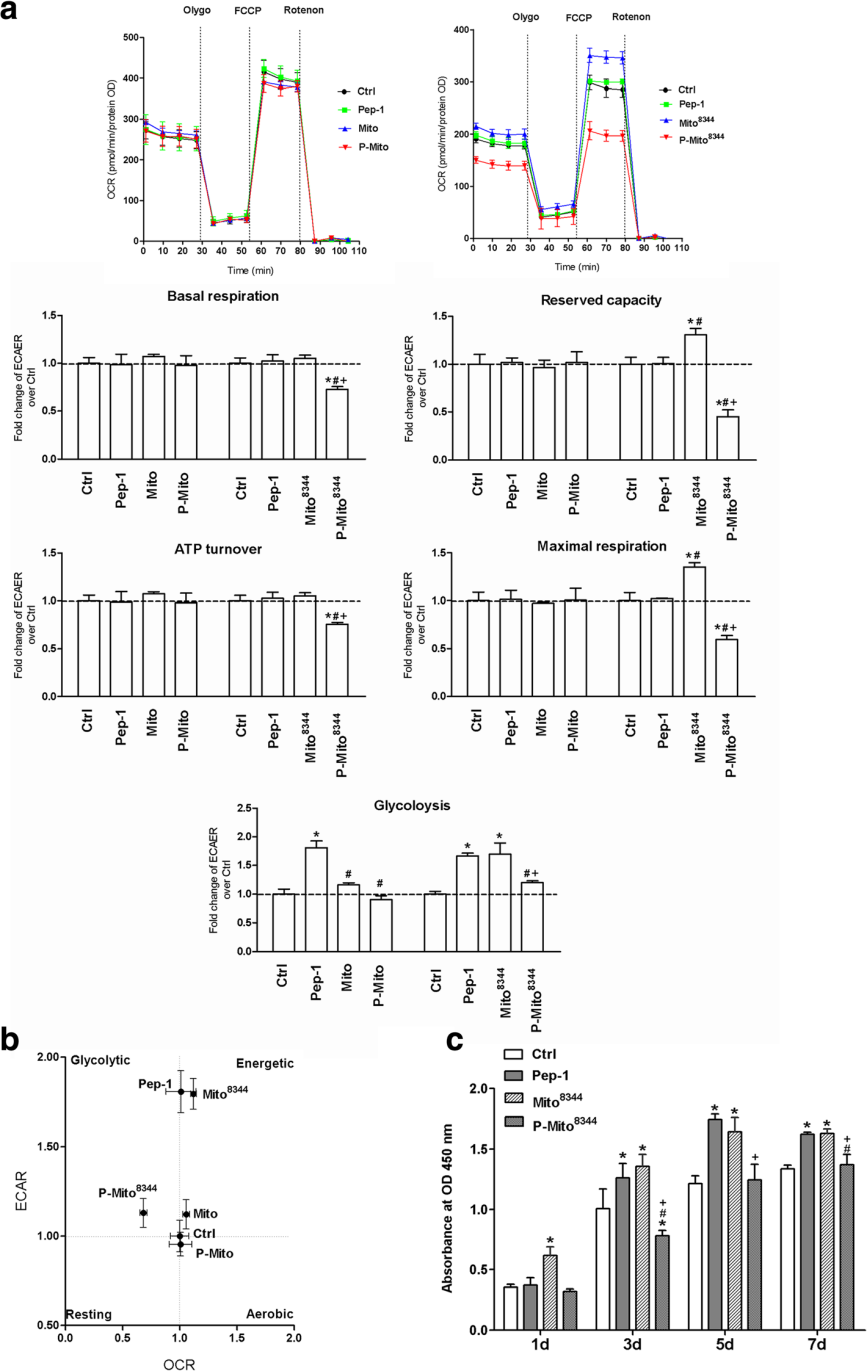
Effect of internalized mitochondrial function on the regulation of energy metabolism and cell viability in MCF-7 cells. Normal mitochondria (Mito) and dysfunctional mitochondria with the mitochondrial DNA (mtDNA) A8344G mutation (Mito^8344^) were isolated from normal and myoclonic epilepsy with ragged-red fibres (MERRF) syndrome cybrid cells, respectively. After cells received mitochondrial transplantation with (P-Mito and P-Mito^8344^) or without Pep-1 modification (Mito and Mito^8344^) for 3 days, mitochondrial basal respiration and glycolysis were measured by monitoring consumption rates (OCRs) and extracellular acidification rates (ECARs) in real time, respectively (**a**). Mitochondrial ATP turnover, maximal respiration and reserved capacity were analysed by individually calculating the OCR response to mitochondrial ATP synthase inhibitor Oligomycin (Olygo, 1 μM), an uncoupler of FCCP (0.3 μM) and complex I inhibitor of Rotenone (1 μM). Quantifications of the OCR and ECAR were normalized to the quantity of cells (pmoles/min/protein OD) (**a**). The profile of metabolic phenotypes according to respiration and glycolysis showed metabolic reprogramming by mitochondrial transplantation (**b**). The effect of the Mito^8344^ or P-Mito^83344^ treatments on cell viability was evaluated by WST-1 proliferation assay on days 1, 3, 5 and 7 (**c**). * *p* < 0.05, difference relative to the control (Ctrl) group. # *p* < 0.05, difference relative to the Pep-1 group. + *p* < 0.05, difference between the Mito and P-Mito groups

There were no significant differences in mitochondrial metabolism, including basal respiration, ATP turnover and maximal respiration, or glycolysis in the Mito and P-Mito groups (Fig. 7a). However, transplantation of dysfunctional mitochondria with the mtDNA A8344G mutation (Mito^8344^) instead of normal mitochondria resulted in a comprehensively significant decrease in oxidative phosphorylation (OXPHOS) in the Pep-1-labelled Mito^8344^ (P-Mito^8344^) group relative to the control group, in contrast to the Mito^8344^ group, which had higher inductions of mitochondrial maximal respiration, reserved capacity and glycolysis (Fig. 7a). This finding indicated that the regulation by the P-Mito treatment, but not by the Mito treatment, occurred in a mitochondrial function-dependent manner. The distinct regulation of the metabolic phenotype profile is also shown in Fig. 7b, where shifting the phenotype towards more energetic and glycolytic phenotypes was observed in the Mito^8344^ and P-Mito^8344^ groups, respectively, relative to the control group. This was mainly due to individual increases in glycolysis and inhibition of mitochondrial metabolism in the Mito^8344^ and P-Mito^8344^ groups compared with each normal mitochondrial treatment group. Moreover, inhibition of cell proliferation was abolished by replacing normal mitochondria with dysfunctional mitochondria (Fig. 3b and Fig 7c). Cell proliferation on day 7 in the Mito^8344^ group was significantly higher than that in the P-Mito^8344^ group, even higher than that in the control group and equivalent to that in the Pep-1 group (Fig. 7c). Compared to the control group, the Pep-1 groups showed a dominant increase in glycolysis rather than increases in the regulation of mitochondrial respiration (Fig. 7a) and cell proliferation (Fig. 7c).

## Discussion

The present study demonstrated that mitochondrial transplantation via simple co-culture and Pep-1-mediated mitochondrial delivery successfully impaired MCF-7 breast cancer cell viability, manifested as inhibition of cell proliferation, induction of caspase-independent and AIF-mediated cell apoptosis and increased chemotherapeutic sensitivity. Furthermore, the mechanism was related to the regulation of mitochondrial fusion and parkin machinery under the P-Mito and Mito treatments, respectively. The feasibility of antitumour growth by mitochondrial transplantation was further approved in vivo. Tumourigenesis in advanced severe immunodeficiency (ASID) mice (NOD.Cg-Prkdc^scid^ Il2rg^tm1Wjl^/YckNarl) further confirmed the use of another human breast cancer cell line MDA-MB-231 (Additional file 1: Supplementary methods). Compared with the non-treated control cells and in contrast to the Pep-1-treated cells, the Mito- and P-Mito-treated cells showed consistent suppression of tumourigenicity, as revealed by significant reductions in tumour volume (mm^3^) (Additional file 2: Figure S1a), significant reductions in tumour weight (g) (Additional file 2: Figure S1b), significant inductions of apoptotic chromatin condensation by transmission electron microscopy (TEM) (Additional file 2: Figure S1c and d) and DNA fragmentation by terminal deoxynucleotidyl transferase dUTP nick end labeling (TUNEL) assay (Additional file 2: Figure S1e and f). Furthermore, intriguingly, none of the treatments, namely, Mito, P-Mito and Pep-1, affected the cell viability of the human breast epithelial cell line MCF-10A, a non-tumourigenic breast epithelial cell line (Additional file 3: Figure S2). Thus, the mitochondrial transplantation-induced anticancer effect was not caused by the toxicological effects of foreign mitochondrial organelles and even increased antioxidant enzyme levels, consequently delaying oxidative stress. The consistent observation was made in the MDA-MB-231 breast tumours of ASID mice, based on staining with a marker of oxidative stress (8-hydroxy-2′-deoxyguanosine (8-OHdG) (Additional file 1). There were marked decreases in the 8-OHdG levels, represented by oxidative DNA damage, in both the Mito and P-Mito groups relative to the control group, but there was no difference between the Pep-1 and control groups (Additional file 4: Figure S3).

Mitochondrial transplantation has recently been regarded as a potential therapeutic for mitochondrial diseases [25–27]. Isolated mitochondria can be passively internalized via cytoskeleton-dependent macropinocytosis [20], but intake efficiency varies according to cell properties [15]. Previously, we showed that an obstacle of spontaneous mitochondrial uptake in vitro in cybrid cell lines and primary cells from mitochondrial disorders such as MERRF or MELAS [16–18] was related to cytoskeletal disruption in these diseased cells [15]. Thus, mitochondrial carriers are essential to increase the incorporation efficiency in specific diseased cells [18, 19] and assist in preventing mitochondrial inactivation caused by a prolonged exposure time to the extracellular environment [26]. In this study, in contrast to the abovementioned cell types, we found that the MCF-7 human breast cancer cell line could incorporate naked mitochondria suspended in medium without Pep-1 conjugation, as previously shown by Elliott R.L. et al. [13]. However, the partial mitochondrial adherence to the cell membrane led to less uptake under these conditions than by PMD. Previously, we showed that the PMD-mediated recovery of mitochondrial function showed dose-dependent regulation in mitochondrial disease cells [17], and treatments that exceeded a dosage threshold induced cell toxicity instead of a therapeutic effect [17]. Thus, the relationship between the mitochondrial intake amount and treatment effectiveness needs further study in cancer cells.

A balanced and tubular mitochondrial network is required for Bax-dependent mitochondrial outer membrane permeabilization (MOMP) after terminal ER stress or chemotherapeutic drug treatment [28]. As a result of MOMP, AIF was released from the mitochondria into the cytosol and then translocated into the nucleus to induce caspase-independent apoptosis caused by chromatin condensation and DNA fragmentation [29]. Herein, we lack the data of AIF expression at initiation of apoptosis after 6–24 h of mitochondrial treatment but release of soluble intermembrane space proteins has been considered as an early event during apoptosis and occurred prior to activation of caspase and cell shrinkage in late apoptosis [30]. Although more insight of apoptotic pathway is needed to clarify due to the time frames, it still could explain the existence of AIF nuclear translocation in treated MCF-7 cells with an interconnected mesh-like mitochondrial network (tubular or elongated morphologies). Thus, the mechanism of mitochondrial transplantation-mediated cell apoptosis was not only related to an increase in AIF nuclear translocation in regulating DNA fragmentation but also related to sustained mitochondrial fusion [31]. This hypothesis was supported by a gradual increase in the number of research studies. The human melanoma cell line A375 was susceptible to anticancer drug-induced apoptosis via an increase in Drp-1-inbitor-induced mitochondrial fusion [28]. Mitochondrial fusion also suppressed pancreatic cancer growth [32]. Moreover, the persistence of mitochondrial hyperfusion induced replication stress and serious chromosomal instability during mitosis to impair cancer growth [33]. Additionally, prolonged mitochondrial fusion caused mitochondrial bridges between daughter cells, resulting in defective cytokinesis, an unequal distribution of mitochondria, incorrect segregation of chromosomes, and aneuploidy [33]. Therefore, the observation that significant decreases in the mitochondrial fusion proteins OPA1 and MFN2 but not the mitochondrial fission protein Drp1 occurred in MCF-7 cells compared to the normal human mammary MCF-10A epithelial cell line is reasonable (Additional file 5: Figure S4; Additional file 1), but it needs more detailed proof to clarify the regulation of mitochondrial fusion in different breast cell lines as a basis for therapy.

Although P-Mito induced marked decreases in the levels of fusion proteins OPA1 and MFN2 with a consequent decrease in PINK1-L protein expression, followed by extensive mitochondrial fusion and a consequently elongated morphology, Mito treatment significantly induced parkin protein expression and tubular morphology. However, a consistent reduction in mitochondrial fission protein Drp1 was observed in both mitochondria treatment groups. Drp1, a member of the dynamin family of guanosine triphosphatases (GTPases), functions not only as the key component of the mitochondrial fission machinery but also as a mediator in the plasticity of tumour cells in various internal and external contexts. Under mild stress, inactivation and/or downregulation of Drp1 may have a counteracting effect on tumourigenesis, manifested as effects on metabolic reprogramming, the cell cycle, cell proliferation, cell invasion and cell migration, and can thus be used as a therapeutic approach for cancer treatment [34]. We also agree on the potential of Drp1 over the other implicated proteins for targeted therapy in the context of breast cancer, although the complex interplay between mitochondrial dynamics and cell requirements is paradoxical at every stage of tumourigenesis and reflects the different levels of environmental stress [34]. On the other hand, dramatic upregulation of parkin is associated with inhibition of tumourigenesis under the Mito treatment. Parkin, an E3 ubiquitin ligase, also plays a role in cancers as a putative tumour suppressor and has been shown to negatively regulate the proliferation of breast cancer cells through enhanced expression of cyclin-dependent kinase 6 [35]. Inhibition of parkin expression induces mitochondrial dysfunction and neuronal death in Parkinson’s disease [36] and aberrant metabolism during tumourigenesis [37]. On the other hand, parkin protein in synergy with PINK1 protein is responsible for mitophagy, the selective degradation of damaged mitochondria. Although the role of mitophagy in the mitochondrial transplantation treatment of breast cancer remains unclear, mitophagy is thought to be an onco-suppressor that prevents oncogenic transformation and maintains cellular homeostasis [38].

In this study, we found that both mitochondrial transplantation treatments consistently decreased ROS levels and increased catalase antioxidant enzyme levels in MCF-7 cells, the latter of which was more pronounced in the P-Mito treatment, with an additional increase in SOD2 protein levels. The result was further confirmed in vivo*,* showing that DNA oxidative damage as revealed by 8-OHdG staining was lower in breast tumour tissues generated from cells with mitochondrial transplantation. ROS actions either as a growth promoter or pro-apoptotic agent depend not only on dosage (concentration) but also on the stage of cell apoptosis and the cell type. Sustained production of ROS in MCF-7 mainly activates survival signalling, facilitates oestrogen unresponsiveness, increases aggressive growth potential, and enables resistance to endocrine therapy [39]. Thus, we proposed that increased antioxidant enzymes and reduced ROS levels balanced the oxidative status within the cancer cells towards the normal level and contributed to increased sensitivity of cancer cells to apoptosis during the early stages through elimination of the cell adaptive protection caused by endogenous ROS stimulation. This pathway is different from the common pathway of late cell apoptosis that is induced by the caspase- and ROS-dependent mitochondrial pathways in human breast cancer cells [40]. This result also underscored our finding that mitochondrial transplantation triggers nuclear translocation of AIF at a relatively early stage of apoptosis, before cytochrome c release is induced by mitochondrial damage during late apoptosis [41]. On the other hand, we suggested that the increased levels of the SOD2 and catalase antioxidant enzymes by mitochondrial transplantation could contribute to increased chemotherapy sensitivity and attenuate the cell aggressive phenotype. Supporting evidence has been obtained using mimetics or genetic overexpression of SOD and catalase [42–44] and antioxidant-based dietary supplements in breast cancer cell models and human studies [45].

It is known that the mechanism of action for chemotherapy drug Doxorubicin is attributed mainly to blocking the transcription and replication of nuclear DNA and increasing DNA damage via the inhibition of topoisomerase II activity [46]. As previous mentions, impairment of cancer growth via persistence of mitochondrial hyperfusion also induced replication stress and serious chromosomal instability during mitosis [33]. Presently, the correlation between chemoresistance and mitochondrial dynamic proteins has been shown that OPA1 loss-induced mitochondrial fragmentation tends to cause an increase in resistance to drug treatments in cancers [47]. Thus, we suggest that facilitation of mitochondrial fusion could be related to increase of susceptibility to cell death by chemotherapeutic treatment with Doxorubicin due to the analogous mechanism of genomic DNA interference. Thus, it reflected that P-Mito-treated cells, regardless of whether the cell line was MCF-7 or MDA-MB-231, were more sensitive to Doxorubicin than Mito-treated cells; however, Mito-treated cells were as sensitive to Paclitaxel as P-Mito-treated cells. Paclitaxel is a mitotic blocker by stabilizing microtubules, and parkin overexpression induced by Mito treatment can promote the binding of parkin to microtubules, resulting in a synergistic enhancement of Paclitaxel-induced microtubule assembly and stabilization to accelerate mitotic block and apoptosis in breast cancer [48]. Thus, this study revealed that the effectiveness of chemotherapy in breast cancer cells varies according to the regulatory machinery of mitochondrial transplantation. Otherwise, it is worth mentioning that WST-1 agent we used maybe not accurate way to detect cell proliferation because reaction of WST-1 by mitochondrial dehydrogenases could be interfered by the extra delivered mitochondria. Thus, we confirmed the mitochondrial transplantation-induced cell toxicity by lactate dehydrogenase (LDH)-released assay. Although we found that the detective sensitivity of cell viability using LDH-released assay (Additional file 6: Figure S5) was more sensitive than WST-1 assay (Fig. 3b) with a greater difference of cell death-induced absorbance, the consistent result of obvious cell impairment occurred at 5th and 7th day of culture was not affected by the different methods in this study.

Transmitochondrial cybrids of breast cancer cells with non-cancerous mitochondria can reverse the malignancy of metastatic breast cancer through inhibition of several oncogenic pathways and suppression of tumourigenesis in nude mice [49]. Herein, we demonstrated similar results in the MCF-7 breast cancer cell line and in an animal model via different approaches of mitochondrial transplantation. These results consistently highlight the prevailing notion of the more decisive role of mitochondria than nuclei in regulating tumourigenesis in breast cancers [49, 50]. The mitochondrial regulation of tumours is thought to be closely related to mitochondrial dysfunction (Warburg effect) and dysregulated energy metabolism, especially the latter, which has been widely studied in recent years. In this study, using transplantation of dysfunctional mitochondria caused by mtDNA mutation, we showed that an increase in energy demand from glycolysis in the Mito^8344^ treatment was more effective in restoring cancer growth than impairment of mitochondrial respiration in the P-Mito^8344^ treatment. Thus, breast cancer is not a mitochondrial disease but can be more accurately called a mitochondrial metabolic disease. We agree that the contributions of mtDNA mutations to tumourigenesis are more dependent on the modulation of energetic/glycolytic metabolic profiles than on the direct inhibition of mitochondrial function, as shown in a previous study [51]. On the other hand, the results also revealed the distinct machinery of the Mito and P-Mito treatments against breast cancer and that the P-Mito treatment actions, but not those of the Mito treatment, occurred through mitochondria function-dependent regulation.

## Conclusion

Treatment of breast cancer using mitochondrial transplantation is not an innovative concept, but this study takes the lead in conducting a comprehensive analysis of mitochondrial function, including mitochondrial dynamics, oxidative stress and metabolism, and comparing the effects of different transplant routes on treatment outcomes. Mitochondrial transplantation can promote apoptosis and inhibit MCF-7 cell proliferation by inducing the nuclear translocation of AIF without affecting mitochondrial function, while reducing cellular oxidative stress and drug resistance. The machinery can be associated with the increase of parkin protein and mitochondrial dynamic proteins as well as reduced fragmented mitochondria. The replacement of normal mitochondria with dysfunctional mitochondria in the transplantation eliminated the original inhibition of cancer growth by different mechanisms, and an increase in glycolysis in the Mito^8344^ group was more effective in restoring cancer cell growth than inhibition of mitochondrial respiration in the P-Mito^8344^ group.

## Additional files

Additional file 1: Supplementary methods. (DOCX 20 kb)

Additional file 2: **Figure S1** Effect of mitochondrial transplantation on MDA-MB-231 cell tumourigenesis in vivo. After 3 days of treatment, the cells were injected into the fat pads of the fourth pair mammary glands of eight-week-old female advanced severe immunodeficiency (ASID) mice to observe in vivo tumourigenesis after 20 days of injection. The left and right breasts of each mouse were randomly selected to receive injections of different groups of cells, and each group had eight graft replicates (scale bar = 1 cm) (a). The volumes of the subcutaneous breast tumours in the mice were calculated with a 3D laser scanning device (a). After sacrifice, the tumours were weighed (b) and analysed by transmission electron microscopy (TEM) to observe the apoptotic death of tumour cells (c). Tumour apoptosis and DNA gragmentaion were determined by quantification of chromatin condensation in the cellular nucleus (N) (c, d) and terminal deoxynucleotidyl transferase dUTP nick end labeling (TUNEL) assay (e, f). * *p* < 0.05, difference relative to the control (Ctrl) group. # p < 0.05, difference relative to the Pep-1 group. + p < 0.05, difference between the Mito and P-Mito groups (TIF 4274 kb)

Additional file 3: **Figure S2** Cell viability of the MCF-10A human breast epithelial cell line after 3 days of treatment. Cell viability was evaluated by WST-1 proliferation assay on days 1, 3, 5 and 7 (b). (TIF 333 kb)

Additional file 4: **Figure S3** Expression of 8-hydroxydeoxyguanosine (8-OHdG) in breast tumours. Twenty days after the injection of treated MDA-MB-231 cells, the breast tumours were used to evaluate and quantify the levels of 8-OHdG, a biomarker of oxidative damage, by an immunohistochemical staining. * p < 0.05, difference relative to the control (Ctrl) group. # p < 0.05, difference relative to the Pep-1 group (TIF 4773 kb)

Additional file 5: **Figure S4** Expressed differences in mitochondrial dynamics and biogenesis between the human breast epithelial cell line MCF-10A and the human breast cancer cell line MCF7. The levels of mitochondrial dynamic-related proteins optic atrophy-1 (OPA1), Mitofusin 2 (MFN2), and dynamin-related protein 1 (Drp-1), as well as mitophagy-related proteins, full-length PTEN-induced putative kinase 1 (PINK-1-L) and Parkin, were analysed and quantified (a). Mitochondrial biogenesis was evaluated by analysing the copy number of mitochondrial DNA (mtDNA) relative to that of the β-actin gene (b), and mitochondrial mass was measured with 10-N-nonyl acridine orange (NAO) staining and by flow cytometry (c). * p < 0.05, difference relative to the MCF-10A group (TIF 181 kb)

Additional file 6: **Figure S5** Cell viability of treated MCF-7 cells was evaluated by LDH release assay on days (d) 1, 3, 5 and 7 after 3-day treatments. * p < 0.05, difference relative to the control (Ctrl) group. # p < 0.05, difference relative to the Pep-1 group. + p < 0.05, difference between Mito and P-Mito groups (TIF 370 kb)

## Abbreviations

ASID: Advanced severe immunodeficiency
MELAS: Mitochondrial encephalomyopathy, lactic acidosis, and stroke-like episodes
MERRF: Myoclonic epilepsy with ragged-red fibres
Mito: Mitochondria
Mito^8344^: Mitochondria from MERRF patients with the A8344G mutation
Mito^GFP^: Green fluorescent protein-tagged mitochondria
Mitotransfer: Mitochondria transfer
mtDNA: Mitochondrial DNA
mtROS: Mitochondrial ROS
P-Mito: Pep-1-labelled mitochondria
P-Mito^8344^: Pep-1-labelled mitochondria from MERRF patients with the A8344G mutation
