# Supplementary material for: Mitochondrial transplantation regulates antitumour activity, chemoresistance and mitochondrial dynamics in breast cancer
Source: J Exp Clin Cancer Res. 2019 Jan 23;38:30. doi: 10.1186/s13046-019-1028-z (PMC6343292; doi:10.1186/s13046-019-1028-z)
Supplement: Supplementary file 1 — Supplementary methods. (DOCX 20 kb) [file 13046_2019_1028_MOESM1_ESM.docx]

**Supplementary methods**

**Animals**

Seven-week-old female advanced severe immunodeficiency (ASID) mice (NOD.Cg-Prkdc^scid^ Il2rg^tm1Wjl^/YckNarl) were purchased from National Laboratory Animal Center (NLAC), NARLabs, Taiwan. Mice were housed in ventilated cages with autoclaved chow, water and bedding and were maintained under the appropriate environment with a 12-hour light/dark cycle, a temperature of approximately 26°C and a relative humidity of 40 to 60% with food and water ad libitum. All animal treatment protocols were approved by the Institutional Animal Care and Use Committee (IACUC) of Changhua Christian Hospital (CCH-AE-105-019). Following one week of acclimation, mice were ready for cancer cell grafting.

**Tumourigenesis**

After 3-day treatments with Pep-1 alone, mitochondria (Mito) or Pep-1-labelled mitochondria (P-Mito) or no treatment (Control, Ctrl), 1×10^6^ MDA-MB231 cells were suspended in 100 μl of Dulbecco's phosphate-buffered saline (DPBS) and injected randomly into the fat pads of the fourth mammary glands of eight-week-old female ASID mice. The left and right breasts of each mouse were randomly selected to receive injections of different treatment groups, and each group had eight graft replicates. After 20 days of injection, *in vivo* tumourigenesis was evaluated by analysing the volumes of the subcutaneous breast tumours in the mice with a 3D laser scanning device (TumorImager) (Biopticon Corporation; Princeton, NJ, USA) and measuring the tumour weights. Viable tumour tissue was analysed for cell apoptosis and oxidative damage. The apoptotic death of tumour cells in tissues revealed by chromatin condensation in nucleus and DNA fragmentation was observed by transmission electron microscopy (TEM) and terminal deoxynucleotidyl transferase dUTP nick end labeling (TUNEL) assay, respectively. Oxidative DNA damage in tumour tissues was analysed by immunohistochemical staining of 8-hydroxydeoxyguanosine (8-OHdG).

**Cell culture**

MCF10A cells were cultured with the MEGM Bullet Kit (Lonza, Basel, Switzerland) with 100 ng/ml cholera toxin (Sigma) and grown at 37°C in a humidified 5% CO_2_-95% air atmosphere. Cells were detached from tissue culture flasks by digestion with 0.05% trypsin (Gibco/BRL).

**TEM**

Tumour tissues were fixed in 2.5% glutaraldehyde in 0.1 M phosphate buffer (pH 7.2) for 4 h at room temperature. Samples were dehydrated in graded solutions of ethanol for 20 min, infiltrated and embedded into resin to cut ultrathin 70-nm sections (Leica EM UC7, Germany). Sections were viewed by TEM (Hitachi H-7000, Japan). The level of chromatin condensation in the nucleus was calculated by normalizing the condensation area to the total area of the nucleus from each photograph.

**Immunohistochemical staining**

Tumour tissues were post-fixed in 4% paraformaldehyde and embedded in Tissue-Tek OCT medium and cryosectioned to a 5-μm thickness. The tissues were pretreated with 0.3% H_2_O_2_ in distilled water and goat serum to block background staining and incubated overnight at 4°C with monoclonal antibodies against 8-hydroxydeoxyguanosine (8-OHdG, Abcam). The colour was developed with horseradish peroxidase (HRP)-conjugated secondary antibody (Jackson ImmunoResearch Laboratories, Bar Harbour, ME, USA), and a 3,3'-diaminobenzidine tetrahydrochloride (DAB, Abcam) substrate was used to detect the 8-OHdG signal. The immunostained sections were examined by Olympus light microscopy equipped with a computer-controlled digital camera (DP71, Olympus).

**TUNEL assay**

Tumor tissue harvested 20 days implantation were embedded in paraffin and sectioned (5 µm thick). Tissue staining was performed using the DeadEnd™ Colorimetric Apoptosis Detection System (Promega, Madison, WI, USA) to detect DNA fragmentation in the tumour sections according to the manufacturer's protocol. Briefly, the slides incubated with equilibration buffer for 10 minutes were used to treat with 20 µg/ml proteinase K solution for 10-minute. After PBS wash, sections were incubated with TdT enzyme at 37°C for 1 h in a humidified chamber for incorporation of biotinylated nucleotides at the 3′hydroxyl ends of DNA. The slides were incubated in horseradish peroxidase-labeled streptavidin to bind the biotinylated nucleotides followed by detection with stable chromagen DAB. The images on the slides were visualized with an Olympus BX40 light microscope equipped with a computer-controlled digital camera (DP71, Olympus Center Valley, PA, USA). Three slides per group were stained and apoptotic cells were identified by dark brown cytoplasmic staining.

**Lactate dehydrogenase (LDH) release assay**

LDH release was measured with a commercially available LDH assay kit (Pierce LDH Cytotoxicity Assay) as per manufacturer’s protocol. Briefly, 50 µL of sample media were transferred to a 96-well plate in triplicate wells and 50 µL of reaction mixture were added to each sample and incubated for 30 minutes in the dark at room temperature. The reaction was stopped by adding 50 µL of stop solution and mixing by gentle tapping. The absorbance was measured at 490nm and 680nm by a spectrophotometer. The 680nm absorbance value (background) was subtracted from the 490nm absorbance values.
