# Supplementary figures and images for: Mitochondrial transplantation regulates antitumour activity, chemoresistance and mitochondrial dynamics in breast cancer
Source: J Exp Clin Cancer Res. 2019 Jan 23;38:30. doi: 10.1186/s13046-019-1028-z (PMC6343292; doi:10.1186/s13046-019-1028-z)

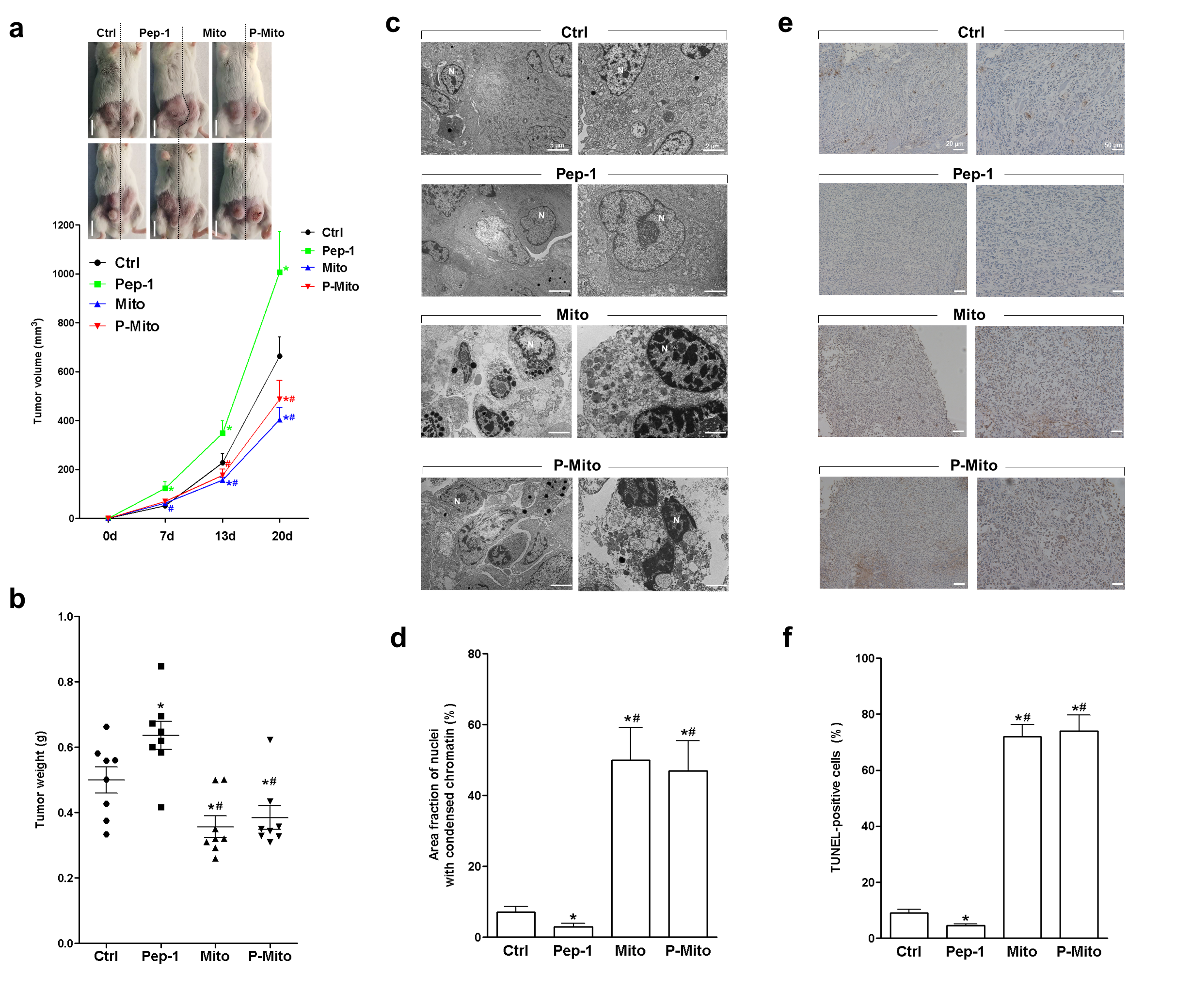

Supplement: Supplementary file 2 — Figure S1 Effect of mitochondrial transplantation on MDA-MB-231 cell tumourigenesis in vivo. After 3 days of treatment, the cells were injected into the fat pads of the fourth pair mammary glands of eight-week-old female advanced severe immunodeficiency (ASID) mice to observe in vivo tumourigenesis after 20 days of injection. The left and right breasts of each mouse were randomly selected to receive injections of different groups of cells, and each group had eight graft replicates (scale bar = 1 cm) (a). The volumes of the subcutaneous breast tumours in the mice were calculated with a 3D laser scanning device (a). After sacrifice, the tumours were weighed (b) and analysed by transmission electron microscopy (TEM) to observe the apoptotic death of tumour cells (c). Tumour apoptosis and DNA gragmentaion were determined by quantification of chromatin condensation in the cellular nucleus (N) (c, d) and terminal deoxynucleotidyl transferase dUTP nick end labeling (TUNEL) assay (e, f). * p < 0.05, difference relative to the control (Ctrl) group. # p < 0.05, difference relative to the Pep-1 group. + p < 0.05, difference between the Mito and P-Mito groups (TIF 4274 kb) [file 13046_2019_1028_MOESM2_ESM.tif]

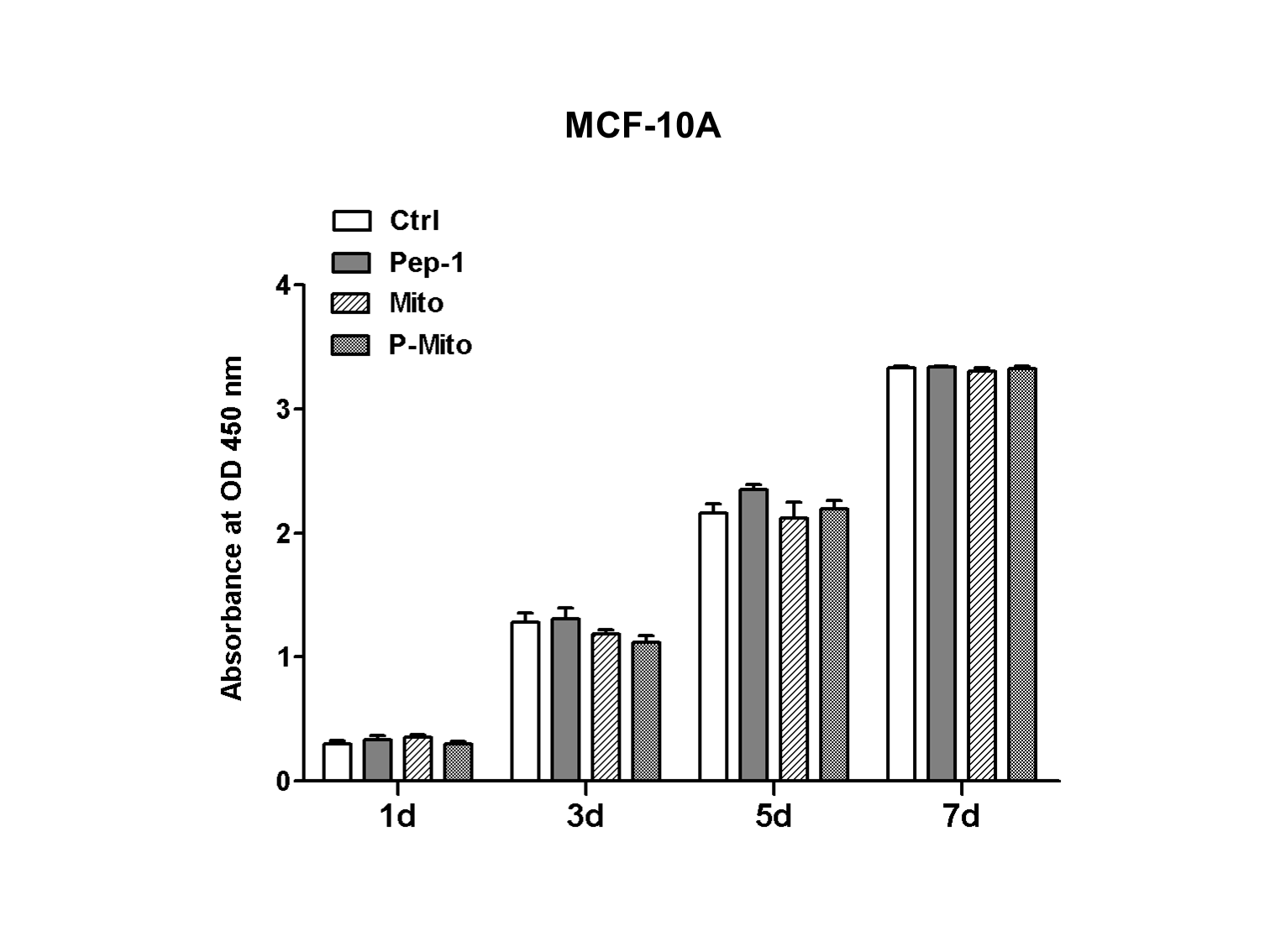

Supplement: Supplementary file 3 — Figure S2 Cell viability of the MCF-10A human breast epithelial cell line after 3 days of treatment. Cell viability was evaluated by WST-1 proliferation assay on days 1, 3, 5 and 7 (b). (TIF 333 kb) [file 13046_2019_1028_MOESM3_ESM.tif]

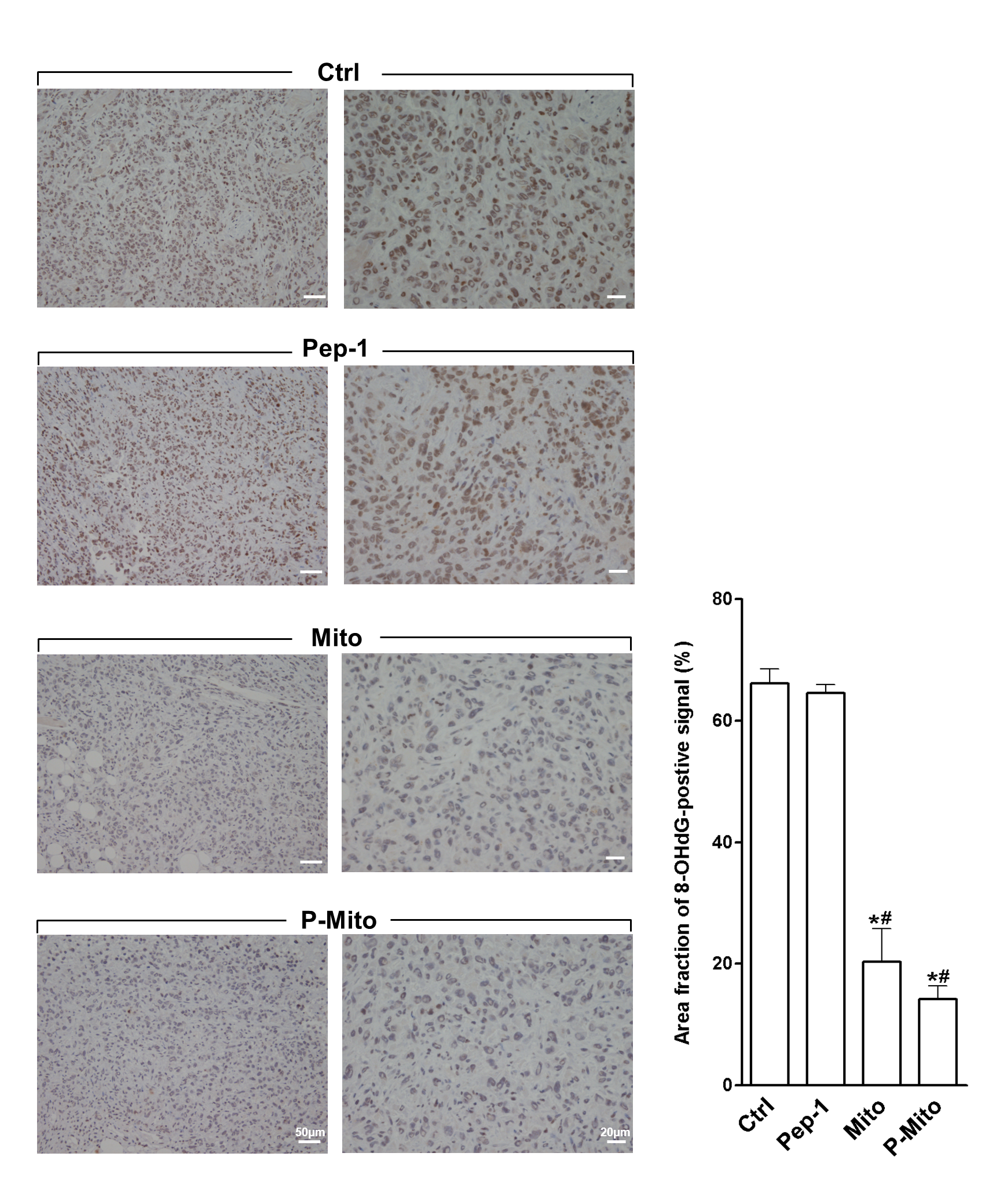

Supplement: Supplementary file 4 — Figure S3 Expression of 8-hydroxydeoxyguanosine (8-OHdG) in breast tumours. Twenty days after the injection of treated MDA-MB-231 cells, the breast tumours were used to evaluate and quantify the levels of 8-OHdG, a biomarker of oxidative damage, by an immunohistochemical staining. * p < 0.05, difference relative to the control (Ctrl) group. # p < 0.05, difference relative to the Pep-1 group (TIF 4773 kb) [file 13046_2019_1028_MOESM4_ESM.tif]

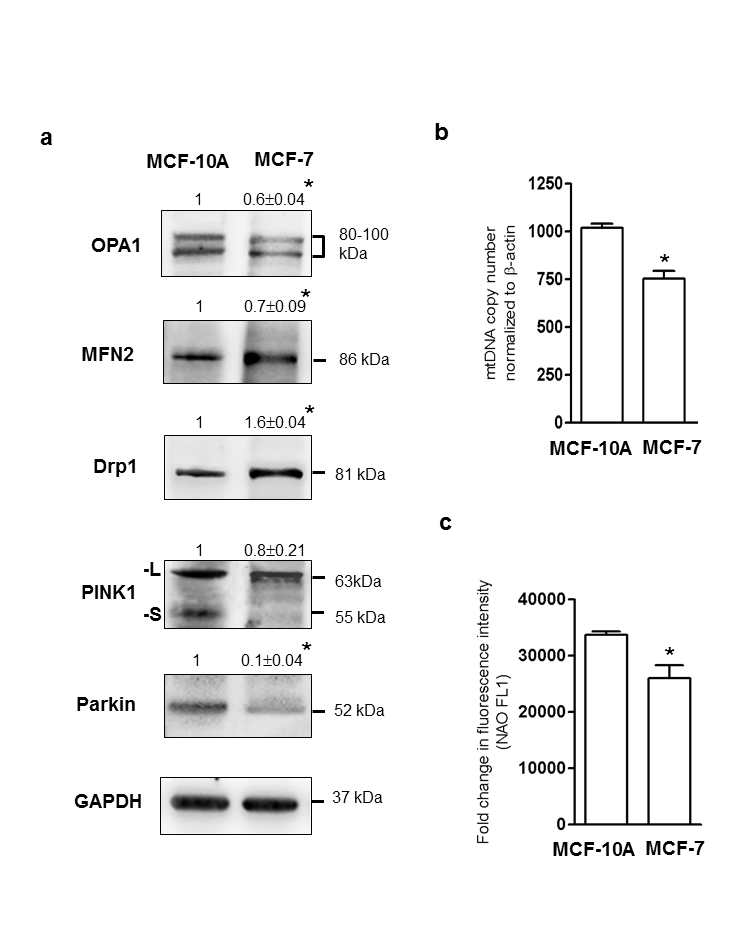

Supplement: Supplementary file 5 — Figure S4 Expressed differences in mitochondrial dynamics and biogenesis between the human breast epithelial cell line MCF-10A and the human breast cancer cell line MCF7. The levels of mitochondrial dynamic-related proteins optic atrophy-1 (OPA1), Mitofusin 2 (MFN2), and dynamin-related protein 1 (Drp-1), as well as mitophagy-related proteins, full-length PTEN-induced putative kinase 1 (PINK-1-L) and Parkin, were analysed and quantified (a). Mitochondrial biogenesis was evaluated by analysing the copy number of mitochondrial DNA (mtDNA) relative to that of the β-actin gene (b), and mitochondrial mass was measured with 10-N-nonyl acridine orange (NAO) staining and by flow cytometry (c). * p < 0.05, difference relative to the MCF-10A group (TIF 181 kb) [file 13046_2019_1028_MOESM5_ESM.tif]

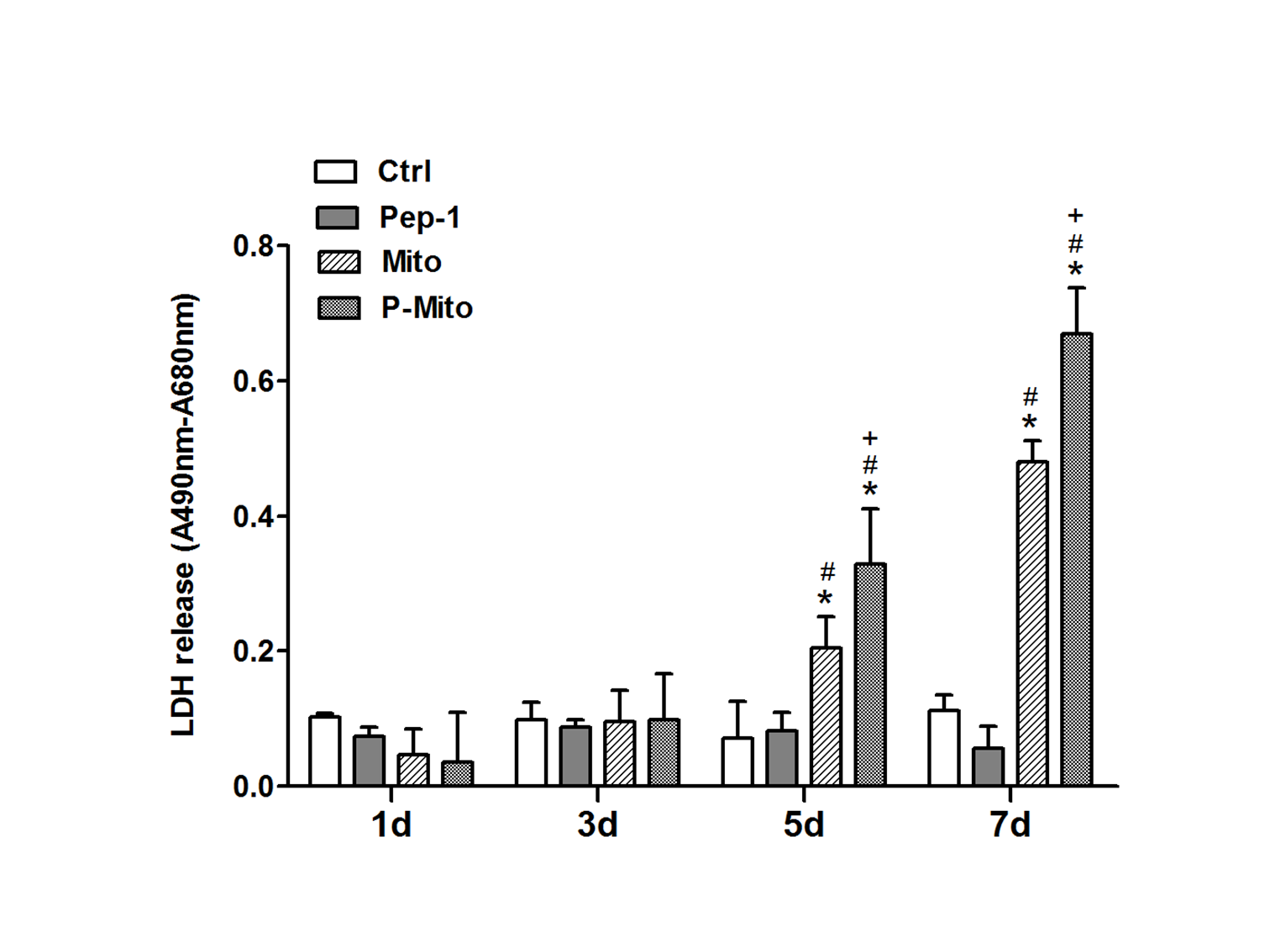

Supplement: Supplementary file 6 — Figure S5 Cell viability of treated MCF-7 cells was evaluated by LDH release assay on days (d) 1, 3, 5 and 7 after 3-day treatments. * p < 0.05, difference relative to the control (Ctrl) group. # p < 0.05, difference relative to the Pep-1 group. + p < 0.05, difference between Mito and P-Mito groups (TIF 370 kb) [file 13046_2019_1028_MOESM6_ESM.tif]
